# Revising NHE‐1: From Cardiac Homeostasis to Heart Failure and Future Drug Development

**DOI:** 10.1002/cbf.70291

**Published:** 2026-09-01

**Authors:** Vasileios Bouratzis, Nikoleta Douskou, Nikolaos P. Tzavellas, Yannis V. Simos, Petros Bozidis, Konstantinos I. Tsamis, Georgios S. Markopoulos, Dimitrios Peschos, Lampros Lakkas

**Affiliations:** ^1^ Second Department of Cardiology University of Ioannina Medical School Ioannina Greece; ^2^ Department of Physiology, Faculty of Medicine, School of Health Sciences University of Ioannina Ioannina Greece; ^3^ Nanomedicine and Nanobiotechnology Research Group University of Ioannina Ioannina Greece; ^4^ Microbiology Department, School of Health Science, Faculty of Medicine University of Ioannina Ioannina Greece

## Abstract

NHE‐1 is a Na^+^/H^+^ exchanger that receives phosphorylation signals, binds calmodulin and responds to neurohormonal input from angiotensin II, endothelin‐1, and adrenergic pathways. In cardiac myocytes, NHE‐1 maintains pH homeostasis and couples to Na^+^/Ca^2+^ exchange and mitochondrial ion handling. During heart disease sustained activation drives intracellular Na^+^ accumulation, promoting Ca^2+^ overload and mitochondrial dysfunction. Oxidative stress then creates amplifying cycles that activate signaling pathways resulting to arrhythmias and fibrosis. Clinical trials failed despite preclinical promise, due to a variety of false experimental factors. SGLT2 inhibitors appear to modulate NHE‐1 indirectly through metabolic reprogramming and hemodynamic effects rather than direct blockade. Current approaches use structural data to target regulatory sites and phosphorylation‐dependent conformational states instead of the transport pore. Translation to patients will require biomarkers identifying pathological hyperactivity and better patient stratification methods. Here, we try to review NHE‐1 structure, regulation, and physiology that may influence research on future drug development.

## Highlights

- NHE‐1 regulates cardiac pH and Na^+^ homeostasis through phosphorylation and calmodulin binding.
- Sustained NHE‐1 activation drives Na^+^/Ca^2+^ overload, mitochondrial dysfunction, and arrhythmias
- Classical NHE‐1 inhibitors failed clinically due to poor selectivity and off‐target toxicity
- SGLT2 inhibitors modulate NHE‐1 indirectly through metabolic and hemodynamic mechanisms
- Regulatory‐site targeting and tissue‐specific delivery offer new therapeutic strategies

## 1 Introduction

Over 64 million people worldwide live with heart failure, a number that continues rising as populations age and survival from acute events improves [1]. Sodium and calcium mishandling drives importantly the underlying pathophysiology [2]. NHE‐1 overactivity elevates intracellular sodium, which then promotes calcium entry through reverse‐mode Na^+^/Ca^2+^ exchange [3, 4]. This sodium‐calcium cascade contributes to maladaptive remodeling and accelerates disease progression. In animal models, NHE‐1 blockade reduces hypertrophy and structural damage, suggesting therapeutic potential in humans [5]. Given the consistent calcium overload observed in failing myocardium, interventions targeting upstream sodium transporters warrant investigation [6].

NHE‐1 is a ubiquitous membrane protein that exchanges intracellular protons for extracellular sodium, maintaining pH and cellular homeostasis [7]. In the heart, this exchanger presents a therapeutic paradox: under normal conditions, it regulates pH, but during stress, it can shift into sustained hyperactivity [2]. This becomes especially flawed during ischemia‐reperfusion, where NHE‐1‐driven sodium uptake leads to calcium overload and myocardial damage [8]. The therapeutic opportunity lies in selective modulation rather than complete inhibition‐ reducing pathological hyperactivity while preserving basal housekeeping functions could protect against cardiac hypertrophy and heart failure [9, 10].

Early enthusiasm stemmed from extensive experimental studies showing NHE‐1 inhibitors could protect myocardium against ischemic injury [11]. However, clinical trials with cariporide and eniporide produced mixed results, with limited cardioprotective benefits and potential side effects [12]. Preclinical data showing myocardial protection remains reproducible across species and experimental models. In addition, NHE‐1 blockade may limit pathological remodeling even when acute infarct reduction is modest, suggesting therapeutic value beyond the original ischemia‐reperfusion paradigm. Current efforts focus on developing more selective inhibitors and identifying the clinical contexts where NHE‐1 hyperactivity is most pathogenic [13, 14].

SGLT2 inhibitors modulate NHE‐1 function, though whether through direct inhibition or indirect effects on protein expression remains controversial [13]. SGLT2i treatment improves cardiac contractility and mitochondrial efficiency by reducing inflammation, oxidative stress, and apoptosis [15]. These findings suggest that predictive models combining clinical features could guide patient selection for NHE‐1‐targeting therapies [16, 17]. NHE‐1 sits at an intersection where metabolic and cardiovascular interventions converge.

## 2 Structure and Regulation of NHE‐1

### 2.1 Structure and Gene

Na^+^/H^+^ exchanger isoform 1 (NHE‐1) is encoded by the SLC9A1 gene and consists of roughly 815 amino acids. While NHE‐1 is found ubiquitously in mammalian tissues, it shows particularly abundant expression in cardiac myocytes. It is the canonical plasma‐membrane, electroneutral NHE responsible for extruding intracellular H^+^ in exchange for extracellular Na^+^, thereby maintaining intracellular pH, cell volume, and sodium homeostasis, functions fundamental to both cardiomyocyte physiology and pathophysiology [18].

**Figure 1:**
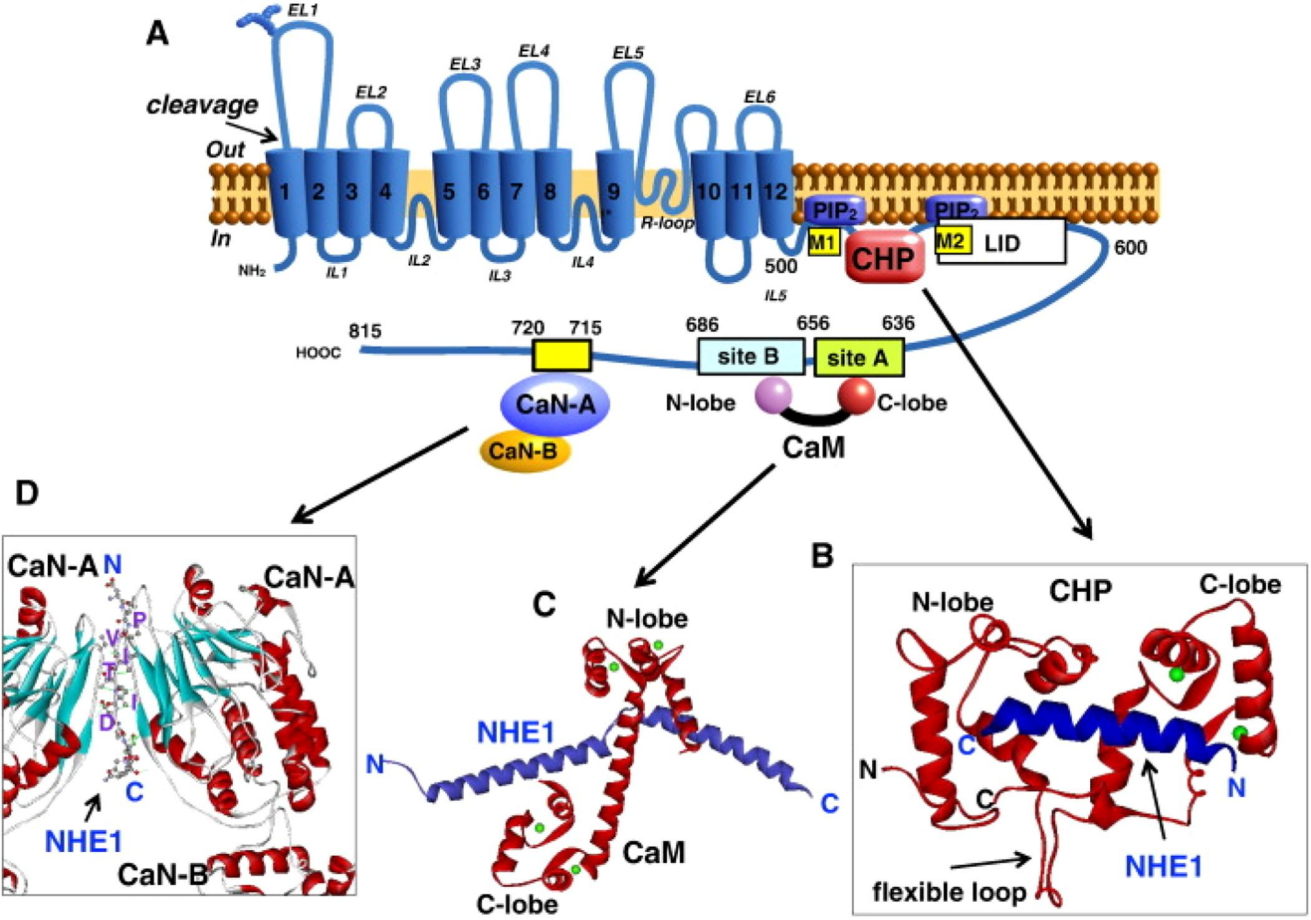
(A) Topology model of NHE1 and 3‐dimensional structure of accessory proteins in complex with NHE1‐derived peptides. NHE1 is separated into 2 large domains, the N‐terminal transmembrane domain (~ 500 residues) containing membrane‐spanning helices and the C‐terminal cytoplasmic domain (~ 300 residues) interacting with various cytosolic factors. Binding sites of calcineurin B homologous protein (CHP), calmodulin (CaM), and calcineurin A (CaN‐A) in the NHE1 cytoplasmic domain have been indicated. Structural models for the CHP2–NHE1 peptide (B) and CaM–NHE1 peptide (C) were created from protein data (pdb ID: 2BEC[65] and 2YGG[79], respectively). The green spheres indicate Ca^2 +^ ions. (D) The structure of the CaN–NHE1‐peptide complex was modeled using PD‐FAMS Ligand & Complex [141] by replacing PHPVIVITGPHEE with the NHE1‐derived sequence DLPVITIDPASPQ based on the reference structure (pdb ID: 2P6B) [137]. Only part of the overall structure (2CaN‐A/2CaN‐B/NHE1‐peptide complex) is represented in this figure. NHE1‐derived peptide forming β‐strand (ball‐stick representation) is sandwiched by β‐sheets of 2 CaN‐A molecules. LID, lipid‐interacting domain; PIP_2_, phosphatidylinositol 4,5‐bisphosphate; M1 and M2, PIP_2_‐binding sites. Reproduction with permission from *Lei M, Salvage SC, Jackson AP, and Huang CL‐H (2024) Cardiac arrhythmogenesis: roles of ion channels and their functional modification. Front. Physiol.15:1342761. doi: 10.3389/fphys.2024.1342761*. This is an open access article under the CC BY 4.0 license.

Structurally, NHE‐1 is a modular protein comprising two functionally distinct regions. The first roughly 500 amino acids comprise the N‐terminal transmembrane (TM) domain, which forms the ion‐translocation machinery. This region contains multiple α‐helices that span the membrane, commonly represented as twelve TM segments [19]. This domain mediates the alternating‐access transport of Na^+^ and H^+^ across the sarcolemma membrane. The C‐terminal cytosolic tail (approximately 300 residues) is a large regulatory domain that integrates multiple signaling inputs through sites of phosphorylation, protein‐protein interaction, and calmodulin (CaM) binding. Structural and cryo‐electron microscopy analyzes, supported by biochemical studies, confirm this two‐domain architecture and highlight the conformational flexibility that underlies allosteric regulation [20] (Figure 1).

In cardiac myocytes, NHE‐1 localizes predominantly to the sarcolemma and the transverse‐axial tubular system, where it forms part of the highly organized ion‐handling microdomains. This strategic localization enables NHE‐1 to respond dynamically to local pH changes, integrate with Na^+^/Ca^2+^ exchanger (NCX) activity, and influence intracellular Ca^2+^ and contractile signaling. By coupling to cytoskeletal and scaffolding proteins, NHE‐1 contributes to both mechanical and biochemical signaling at the plasma membrane, positioning it as a structural and functional nexus in the cardiomyocyte [21].

### 2.2 Molecular Regulation

#### 2.2.1 pH Sensing and Intracellular Activation of NHE‐1

Intracellular acidification directly activates NHE‐1, creating a feedback mechanism that helps restore intracellular pH balance. This pH sensitivity arises from dynamic structural interactions between the ion‐translocation domain and the cytosolic regulatory tail. Protonation of histidine and other titratable residues within regulatory regions triggers allosteric conformational changes, which in turn modify both the maximal transport capacity and the apparent affinity for intracellular H^+^. Consequently, the cytosolic tail operates as a pH sensor, coupling metabolic acid generation to activation of the exchanger [22].

#### 2.2.2 Regulation of NHE‐1 by Phosphorylation

Phosphorylation represents a central regulatory mechanism governing NHE‐1 activity, with multiple kinases targeting specific serine and threonine residues within the C‐terminal cytosolic domain to modulate the exchanger's responsiveness to extracellular signals. Among regulators, ERK1/2 and its downstream effector p90 ribosomal S6 kinase (p90RSK) phosphorylate residues such as Ser703, Ser770, and Ser771, thereby enhancing transporter activity and altering its sensitivity to intracellular pH. Additional kinases, including protein kinase C (PKC), Akt, and Ca^2+^/calmodulin‐dependent kinase II (CaMKII), further contribute to context‐dependent regulation, while serine/threonine phosphatases such as calcineurin counteract these effects through dephosphorylation, ultimately reducing NHE‐1 activity [23]. These phosphorylation events not only determine basal activity but also govern stimulus‐dependent activation during ischemia, receptor stimulation, or mechanical stress.

#### 2.2.3 Integration of Ca^2+^/Calmodulin Signaling and Regulatory Crosstalk

The cytosolic tail of NHE‐1 contains calmodulin (CaM)‐binding domains that confer sensitivity to intracellular Ca^2+^ levels, with Ca^2+^/CaM binding inducing conformational changes that affect both transporter kinetics and protein–protein interactions. Structural and biophysical evidence indicates that CaM can associate with the exchanger in multiple modes depending on its phosphorylation state, allowing NHE‐1 to integrate signals from intracellular pH, Ca^2+^, and kinase activity. Importantly, phosphorylation of specific serine residues can reduce CaM binding affinity, thereby modulating NHE‐1 activity in a context‐dependent manner [24]. Through this coordinated regulation, NHE‐1 functions as a versatile signaling hub responsive to metabolic, ionic, and neurohormonal cues characteristic of the cardiac environment, and although these intrinsic mechanisms define its regulatory properties, its overall activity in cardiac tissue is ultimately shaped by extracellular neurohormonal signals [24] (Table 1).

**Table 1:** Multi‐level regulation of NHE‐1 Activity. NHE‐1 activity is regulated through multiple converging mechanisms that integrate metabolic, ionic, and neurohormonal inputs.

Regulatory mechanism	Stimulus/Trigger	Molecular sensor	Target domain	Structural change	Functional consequence	References
pH sensing	Intracellular acidosis	Titratable residues (His, others)	TM domain + C‐terminal tail	Allosteric rearrangement	Increased Vmax; altered H^+^ affinity	[22]
Phosphorylation (activation)	Hormones, stress, ischemia	ERK1/2, p90RSK, PKC, Akt, CaMKII	C‐terminal tail (Ser703, Ser770, Ser771)	Relief of autoinhibition	Enhanced activity; shifted pH sensitivity	[23]
Dephosphorylation (inhibition)	Ca^2+^ elevation	Calcineurin (phosphatase)	C‐terminal tail (multiple sites)	Restoration of autoinhibition	Reduced activity	[23]
Ca^2+^/Calmodulin binding	Elevated intracellular Ca^2+^	Calmodulin	CaM‐binding domains (C‐terminal)	Multiple binding modes	Context‐dependent modulation	[24]
Protein‐protein interactions	Various	Scaffolding proteins, cytoskeletal elements	C‐terminal tail	Membrane microdomain localization	Spatial coupling to NCX, mechanical signaling	[21]

*Note:* These regulatory layers enable context‐dependent modulation, distinguishing adaptive pH homeostasis from pathological hyperactivation. TM, transmembrane; His, histidine; Ser, serine; CaM, calmodulin; NCX, Na^+^/Ca^2+^ exchanger.

### 2.3 Physiological Modulators Focused on Cardiac Function

NHE‐1 operates downstream of multiple neurohormonal signaling pathways that govern cardiac physiology and adaptive remodeling. Under physiological conditions, transient activation by hormones or neurotransmitters supports pH regulation and contractile function.

#### 2.3.1 Angiotensin II and Endothelin‐1 Signaling

Angiotensin II (Ang II) is a major neurohormonal regulator of NHE‐1 activity, particularly under conditions of cardiovascular stress and remodeling. Upon binding to the Ang II type 1 (AT_1_) receptor, Ang II activates multiple intracellular signaling cascades, including PKC and mitogen‐activated protein kinases (MAPKs), such as ERK1/2 [9, 12, 14]. These signaling pathways converge on the C‐terminal regulatory domain of NHE‐1, promoting its phosphorylation and enhancing exchanger activity [22]. The resulting increase in Na^+^ influx leads to intracellular Na^+^ accumulation, which in turn drives Ca^2+^ overload via the Na^+^/Ca^2+^ exchanger, ultimately contributing to contractile dysfunction and pathological cardiac remodeling [14, 22].

Endothelin‐1 (ET‐1) exerts comparable regulatory effects on NHE‐1 through activation of ETA receptors and downstream signaling pathways that partially overlap with those triggered by Ang II. Specifically, ET‐1 stimulates PKC‐ and MAPK‐dependent phosphorylation of NHE‐1, leading to sustained increases in exchanger activity [9, 21]. Importantly, these neurohormonal systems do not function independently; instead, Ang II and ET‐1 act synergistically to amplify NHE‐1 activation in pathological states such as cardiac hypertrophy and heart failure [12]. This coordinated signaling promotes persistent intracellular Na^+^ and Ca^2+^ dysregulation, thereby exacerbating myocardial injury, contractile dysfunction, and structural remodeling.

#### 2.3.2 Adrenergic and Growth Factor Pathways

Besides regulation through the renin‐angiotensin and endothelin systems, adrenergic and growth factor signals also modulate NHE‐1 activity. Adrenergic signaling provides additional regulatory inputs to NHE‐1. α_1_‐Adrenergic receptors activate PKC and MAPK pathways analogous to those of Ang II, while β‐adrenergic signaling indirectly modulates NHE‐1 through cyclic AMP‐dependent mechanisms that influence intracellular Ca^2+^ dynamics and CaM binding. Beyond G‐protein signaling, growth factors such as epidermal growth factor (EGF) and insulin‐like growth factor‐1 (IGF‐1) activate receptor tyrosine kinases that converge on ERK1/2, leading to enhanced NHE‐1 phosphorylation and activation. This convergence underscores NHE‐1's function as a nodal integrator of hormonal, mechanical, and metabolic signals controlling cardiac performance and structural adaptation [21].

## 3 Physiological Role of NHE‐1 in the Heart

### 3.1 Maintenance of Intracellular pH and Na^+^ Balance, and Role in Excitation‐Contraction Coupling

The structural and regulatory features of NHE‐1 described above underpin its diverse physiological roles in the heart. In cardiac myocytes, maintenance of intracellular pH and sodium homeostasis is essential for contractile stability and metabolic integrity. NHE‐1 serves as the principal sarcolemmal H^+^ extrusion pathway, exchanging one intracellular H^+^ for one extracellular Na^+^ [19]. During increased metabolic activity or transient acidosis, NHE‐1 activation accelerates H^+^ efflux, counteracting the fall in intracellular pH. Even small deviations in pH, on the order of 0.2 units, can markedly depress contractile strength by diminishing myofilament Ca^2+^ sensitivity [25]. Thus, precise control of intracellular pH (typically 7.1–7.3) is vital for preserving optimal cross‐bridge cycling, enzymatic function, and excitation‐contraction coupling.

Given this pH‐regulatory role, NHE‐1 couples proton extrusion to Na^+^ entry. The resultant increase in intracellular Na^+^ concentration must be compensated by the Na^+^/K^+^‐ATPase, which maintains the inward Na^+^ gradient that powers the exchanger. In this respect, NHE‐1 functions as a secondary active transporter, driven indirectly by ATP hydrolysis through Na^+^/K^+^‐ATPase activity [19]. While this coupling provides dynamic stability, it operates within narrow constraints. Increased H^+^ extrusion benefits contractile function, yet inadequate Na^+^ clearance can result in cytosolic Na^+^ accumulation.

This Na^+^ influx has downstream consequences: intracellular Na^+^ directly modulates the NCX, NHE‐1 activation indirectly influences Ca^2+^ cycling. Elevated intracellular Na^+^ reduces the driving force for NCX‐mediated Ca^2+^ efflux (forward mode), thereby promoting intracellular Ca^2+^ retention. This enhances sarcoplasmic reticulum (SR) Ca^2+^ loading and can transiently augment contractile amplitude, an effect considered positive inotropic [26]. Simultaneously, the more alkaline cytosol produced by H^+^ extrusion increases myofilament Ca^2+^ sensitivity, reinforcing contractile performance.

### 3.2 Crosstalk with Na^+^/Ca^2+^ Exchanger and Mitochondrial Ion Regulation

Beyond its sarcolemmal functions, NHE‐1 exerts significant influence over mitochondrial ion homeostasis through indirect Na^+^‐dependent mechanisms. The Na^+^ influx mediated by NHE‐1 alters the cytosolic Na^+^ concentration that governs mitochondrial Na^+^ and Ca^2+^ exchange processes. Elevated intracellular Na^+^ enhances mitochondrial Na^+^ uptake via the mitochondrial Na^+^/Ca^2+^ exchanger, which can reduce mitochondrial Ca^2+^ content and alter matrix pH. Conversely, shifts in mitochondrial H^+^ and Na^+^ gradients modulate mitochondrial Na^+^/H^+^ exchange, influencing oxidative phosphorylation, ROS production, and susceptibility to mitochondrial permeability transition pore opening. By modulating cytosolic Na^+^, NHE‐1 thus influences not only sarcolemmal ion balance but also the mitochondrial matrix environment, coupling surface membrane activity to bioenergetic output.

### 3.3 Insights into the Mechanosensitive Activation of NHE‐1 in the Heart

Mechanical stretch, a fundamental stimulus in the beating heart, serves as a potent activator of NHE‐1. Acute increases in preload or afterload rapidly enhance exchanger activity through mechanotransducive signaling pathways. Mechanosensitive activation is thought to involve physical coupling between the cytoskeleton and the C‐terminal regulatory tail, as well as activation of stretch‐sensitive kinases and ROS‐dependent cascades. These inputs converge to increase phosphorylation and CaM‐dependent modulation of NHE‐1, aligning mechanical stress with ionic and metabolic responses [27]. This mechanosensitive behavior enables the heart to maintain intracellular pH and contractility during volume or pressure challenges but also contributes to hypertrophic signaling under sustained mechanical stress. Persistent activation of the NHE‐1‐ERK‐RSK pathway drives early hypertrophic gene expression and cytoskeletal reorganization. Over time, these signaling effects may progress from adaptive hypertrophy toward pathological remodeling.

## 4 NHE‐1 Links Cellular Activation to Cardiac Dysfunction

### 4.1 Cellular Signaling and Molecular Regulation of NHE‐1 in Disease

The MAPK signaling cascade, especially through ERK1/2, represents one of the most frequently examined mechanisms in this context. ERK1/2 responds to hypertrophic stimuli and serves to coordinate both mechanical stress and neurohormonal inputs. Research highlights that ERK1/2 can promote NHE‐1 function through phosphorylation and modulation of its regulatory domain, while the resulting shifts in Na^+^ and Ca^2+^ homeostasis, along with altered redox balance, may enhance MAPK pathway activity, creating a self‐reinforcing loop [2, 28].

Osteopontin (OPN) provides a specific illustration of how NHE‐1 intersects with hypertrophic signaling networks. Abdulrahman et al. demonstrated that increased OPN expression driven by NHE‐1 promotes cardiac hypertrophy via p90RSK, a protein lying downstream of ERK. This finding offers a valuable mechanistic link, tying elevated transporter activity to an established pro‐hypertrophic pathway and to OPN, a mediator implicated in both extracellular matrix remodeling and fibrotic processes [29].

Another central pathway in hypertrophic signaling involves calcineurin and NFAT. Prolonged elevation of intracellular Ca^2+^ activates calcineurin, a phosphatase that enables NFAT transcription factors to enter the nucleus and stimulate expression of hypertrophy‐associated genes. Though the most detailed mechanistic evidence for NHE‐1‐NFAT interactions was established more than a decade ago, reviews on NHE‐1 continue to identify Ca^2+^‐mediated transcriptional regulation, including the NFAT axis, as a critical mechanism through which Na^+^‐induced Ca^2+^ accumulation drives cardiac growth and structural remodeling [2, 9].

### 4.2 Role of NHE‐1 in Cardiac Pathophysiology

#### 4.2.1 NHE‐1 in Cardiac Physiology Beyond pH Regulation

Although the primary function of NHE‐1 is to restore intracellular pH following acidification, its role in cardiac physiology extends far beyond acid–base balance. Cardiomyocytes are continuously exposed to pathological stressors such as ischemia–reperfusion injury, hemodynamic overload, excessive neurohormonal stimulation, oxidative stress, and chronic inflammation. These conditions lead to sustained upregulation of NHE‐1 activity. Because NHE‐1 couples proton extrusion with sodium influx, its overactivation results in intracellular Na^+^ accumulation. This disrupts Ca^2+^ homeostasis, impairs mitochondrial function, alters redox balance, and modulates gene transcription [30].

#### 4.2.2 Mechanisms of NHE‐1 Activation in Disease States

##### 4.2.2.1 Ischemia‐Reperfusion Injury

This interplay between ion fluxes accounts for NHE‐1's continued prominence in current research on cardiac hypertrophy and heart failure. Ischemia‐reperfusion provides a potent stimulus for NHE‐1 overactivation. Multiple signaling pathways modulate exchanger responsiveness during this insult: adrenergic receptors, Ang II, ET‐1, mechanical deformation, and reactive oxygen species amplify NHE‐1 activity substantially beyond the level expected from acidosis alone [9]. Consequently, ischemia‐reperfusion imposes dual activation—acidotic conditions initiate NHE‐1 engagement, yet concurrent stress signaling prolongs this engagement beyond homeostatic need [2].

##### 4.2.2.2 Chronic Cardiac Hypertrophy and Heart Failure

Chronic cardiac hypertrophy and heart failure present a different activation profile. Sustained acidosis plays a lesser role in these settings. Neurohormonal and biomechanical inputs become dominant drivers: persistent sympathetic tone, activation of the renin‐angiotensin‐aldosterone axis, mechanosensitive signaling, and kinase cascades activated by oxidative stress [28]. Such stimuli increase NHE‐1 activity and protein expression, shifting the exchanger from pH regulation toward active participation in pathological remodeling. Phosphorylation, protein‐protein interactions, and allosteric regulation modify NHE‐1 function in response to the disease environment rather than pH fluctuations. In hypertrophy, the exchanger serves as a convergence point for agonists, including Ang II and ET‐1, translating transient signaling inputs into persistent structural alterations [2]. Heart failure compounds these processes through compromised perfusion, episodic ischemia, inflammation, and mitochondrial ROS generation, all of which produce recurrent activation signals [30]. Evidence suggests that once Na^+^ overload and oxidative stress are established, NHE‐1 activation becomes self‐perpetuating [31], contributing to the heart failure phenotype characterized by sodium retention, impaired energetics, and maladaptive signaling.

##### 4.2.2.3 Ionic Dysregulation and Metabolic Consequences

Excessive NHE‐1 activity accumulates intracellular Na^+^, fundamentally altering excitation‐contraction coupling, calcium cycling kinetics, and the ATP cost of contraction [31]. Elevated Na^+^ triggers several pathological cascades. The Na^+^/Ca^2+^ exchanger becomes less efficient at Ca^2+^ extrusion and may operate in reverse mode, importing Ca^2+^ and generating an arrhythmogenic substrate. Ca^2+^ overload extends beyond sarcolemmal effects to disrupt mitochondrial function. Mitochondrial Na^+^/Ca^2+^ exchange governs Ca^2+^ uptake and release by these organelles, modulating dehydrogenase activity and ATP synthesis [32]. Work by Aksentijević and colleagues demonstrated that elevated intracellular Na^+^ redirects cardiac metabolic programs during hypertrophy [32], supporting the concept that Na^+^ operates as a signaling molecule beyond its canonical role in membrane excitability [33]. Consequently, excessive NHE‐1 activity precipitates not only Ca^2+^ dysregulation but also contributes to energy supply‐demand mismatch, promoting ROS generation and reducing contractile reserve.

##### 4.2.2.4 Oxidative Stress and Positive Feedback Mechanisms

Oxidative stress, in turn, creates a positive feedback loop by enhancing NHE‐1 activity. ROS can promote kinase signaling cascades, including MAPK pathways, and modify transporter function along with membrane microdomain organization, thereby lowering the threshold for NHE‐1 activation [9]. This establishes a self‐reinforcing circuit: NHE‐1 activation drives sodium accumulation, which triggers calcium stress that impairs mitochondrial function and elevates ROS generation, which subsequently potentiates further NHE‐1 activation. Contemporary reviews identify this feedback mechanism as a key reason NHE‐1 retains pathophysiological significance even when it is not the primary disease driver [2].

##### 4.2.2.5 Role in Cardiac Fibrosis and Structural Remodeling

Although cardiomyocyte signaling initiates the remodeling process, fibrosis reflects the structural consequences of sustained injury signals. While ionic dysregulation originates primarily in cardiomyocytes, the structural alterations are executed by fibroblasts, which transform into myofibroblasts and synthesize extracellular matrix. Direct investigations linking fibrosis to NHE‐1 in cardiac fibroblasts remain less common than cardiomyocyte‐focused studies, yet emerging data suggest that pH regulation and ion handling in fibroblasts carry mechanistic significance. For example, empagliflozin reduced cardiac fibrogenesis and altered intracellular pH in atrial fibroblasts independently of changes in NHE‐1 protein levels, supporting the broader notion that pH regulatory mechanisms, including potential modulation of NHE‐1 activity, shape fibroblast phenotype [34].

##### 4.2.2.6 NHE‐1 and Arrhythmogenesis

**Figure 2:**
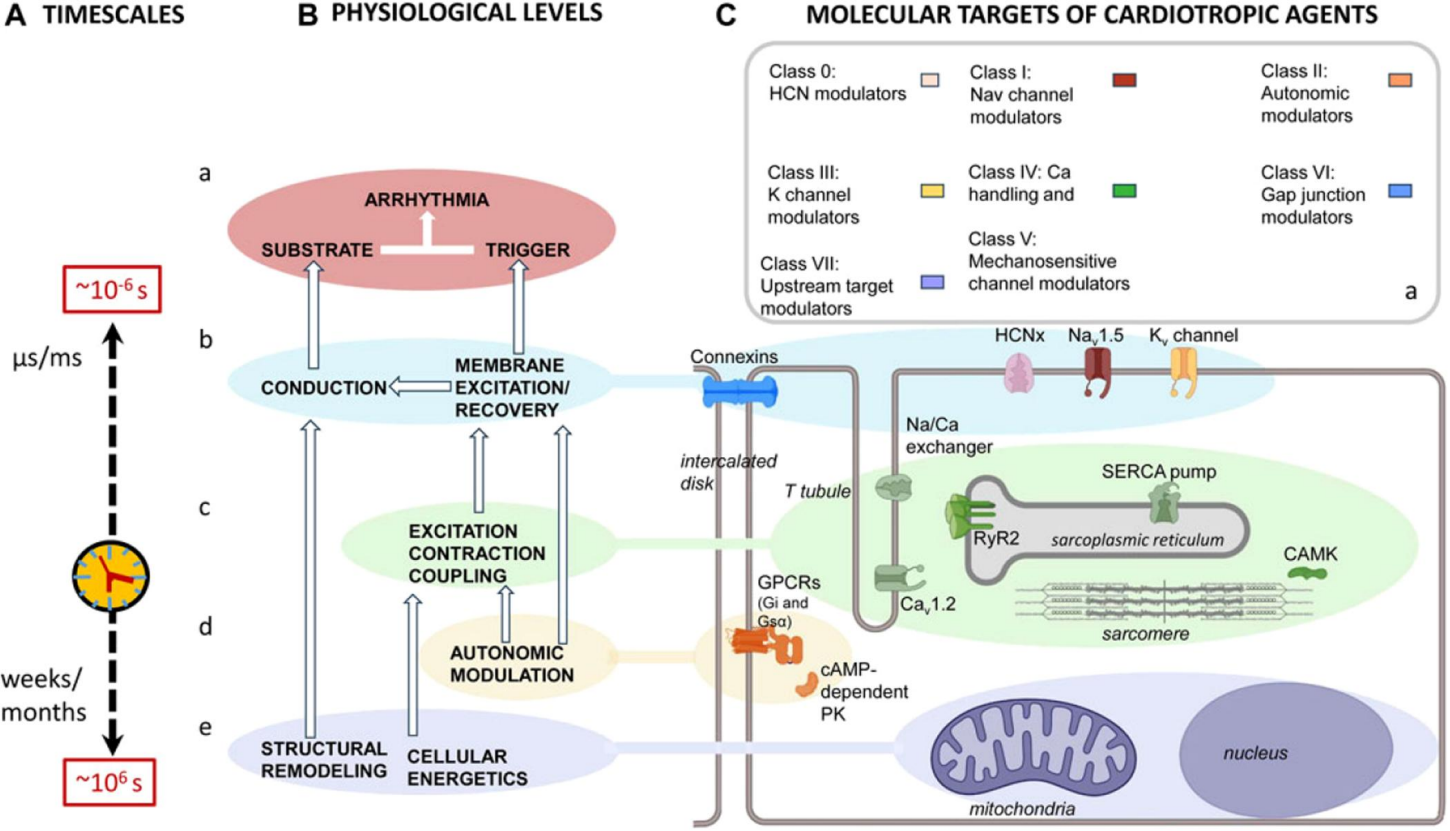
Mapping of physiological mechanisms, molecular targets, and therapeutic agents related to arrhythmia. (A) Pro‐arrhythmic processes taking place over successively longer timescales running from ns/μs, ∼10^–6^ s timescale, molecular events, through ms/s events within each cardiac cycle or multiple cardiac cycles, to hours/days or even week/month, ∼10^6 ^s, timescales resulting from (B) an interleaving hierarchy of physiological processes involving interactions (a) at the molecular/membrane level, interactions between surface membrane ion channels underlying automaticity and AP excitation, propagation and recovery. (b) At the cellular level, excitation contraction coupling processes mediate Ca2+signaling. (c) At the systems level, autonomic inputs and their related G‐protein signaling, modulated by central nervous system circadian rhythms. (d) Longer‐term electrophysiological and structural remodeling effects related to metabolic feedback and other upstream modulators. These map onto (C) recently reclassified (a) anti‐arrhythmic drugs that, in turn, act upon (b–e) the molecular targets mediating these physiological processes. These include (b) surface membrane ion channels contributing inward depolarizing hyperpolarization‐activated cyclic nucleotide gate (HCN); cardiac Na + (Nav1.5) and Ca2+ (Cav1.2) channel, outward K+ channel (Kvx.x), and conducting connexin (CX) mediated currents. (c) Excitation contraction coupling‐related Cav1.2, cardiac ryanodine receptor type 2 (RyR2) and transient receptor potential channel (TRPx), and their regulatory calcium/calmodulin kinase II (CaMKII); (d) autonomic signaling through inhibitory Gi, and stimulatory G proteins, Gs, involving PKA signaling. Longer‐term modulation involves (e) energetic and modeling processes. Reproduction from *Lei M, Salvage SC, Jackson AP, and Huang CL‐H (2024) Cardiac arrhythmogenesis: roles of ion channels and their functional modification. Front. Physiol.15:1342761. doi: 10.3389/fphys.2024.1342761*. This is an open access article under the CC BY 4.0 license.

Disruptions in Na^+^ and Ca^2+^ homeostasis increase susceptibility to arrhythmias. Elevated intracellular Na^+^ impairs Ca^2+^ extrusion via NCX, leading to increased diastolic Ca^2+^ levels and spontaneous Ca^2+^ release from the SR. These events generate transient inward currents through NCX, triggering delayed afterdepolarizations and ectopic activity, particularly in atrial tissue where fibrosis promotes conduction abnormalities and reentry circuits [35] (Figure 2). The late sodium current, though mechanistically distinct from NHE‐1, produces the same consequence: elevated intracellular Na^+^ [36]. This convergence identifies Na^+^ accumulation as a unifying mechanism linking arrhythmia substrates and heart failure pathways [37].

##### 4.2.2.7 Evidence from Human Studies

Human data link enhanced Na^+^ entry to atrial pathology in heart failure. Trum et al examined atrial cardiomyocytes obtained from patients with HFpEF and documented elevated intracellular Na^+^ influx relative to non‐failing control samples [38]. This increase was associated with impaired Ca^2+^ homeostasis and electrophysiological instability. The same investigators evaluated empagliflozin's effects in these human cells, contributing to ongoing discussion about whether SGLT2 inhibitors influence cardiac Na^+^ balance via direct transporter interactions, such as with NHE‐1, or through broader metabolic and ionic remodeling. Independent of the precise mechanism, these observations establish that pathological Na^+^ entry is detectable in diseased human atrial myocardium. Considering the mechanistic coupling between intracellular Na^+^, Ca^2+^ accumulation through NCX, and action potential destabilization, excessive Na^+^ influx may contribute to both atrial contractile impairment and arrhythmic vulnerability in heart failure [38].

Structural remodeling and arrhythmias reinforce each other, as fibrosis and hypertrophy promote reentry while arrhythmias worsen hemodynamics and neurohormonal activation. These responses further stimulate NHE‐1 and MAPK signaling, driving disease progression [38]. Overall, NHE‐1 links ionic imbalance, ischemic injury, and remodeling, contributing to both acute damage and the progression to heart failure.

In addition to its established physiological role, the potential contribution of genetic alterations in NHE‐1 has also been explored. NHE‐1 plays a critical role in maintaining cardiac homeostasis; however, well‐characterized monogenic disorders directly associated with mutations in the SLC9A1 gene are rare [8]. In the cardiovascular system, pathological outcomes are more commonly linked to dysregulation of NHE‐1 activity, including changes in its expression and post‐translational modification, rather than to inherited structural defects [8]. Experimental evidence indicates that impaired NHE‐1 function disrupts intracellular pH control, ion balance, and cell survival pathways, all of which are key processes in cardiac remodeling and heart failure [39]. In addition, emerging data suggest that genetic variation in SLC9A1 may influence disease susceptibility or modulate cardiovascular phenotypes, although this remains to be fully elucidated [39]. Further studies are required to clarify the contribution of NHE‐1 genetic regulation to cardiac pathology and its potential as a therapeutic target.

## 5 Therapeutic Targeting of NHE‐1

The NHE‐1 is a ubiquitous plasma‐membrane transporter that extrudes H^+^ in exchange for Na^+^, thereby regulating intracellular pH, Na^+^ cellular shifting, cell volume, and cytoskeletal signaling. In excitable tissues, especially myocardium, NHE‐1 becomes maladaptive when stimulated by Ι/R, neurohormonal activation, oxidative stress, and mechanical stretch, contributing to intracellular Na^+^ accumulation and secondary Ca^2+^ overload via the Na^+^/Ca^2+^ exchanger. This mechanistic link has kept NHE‐1 on the shortlist of attractive therapeutic targets, despite past disappointments in translation. Contemporary work has reframed NHE‐1 as a context‐dependent effector rather than a constitutive “block‐at‐all‐costs” target, motivating more selective approaches [30, 40].

### 5.1 Pharmacologic Inhibition: Classical Agents (Cariporide, Eniporide) and Trial Outcomes

**Figure 3:**
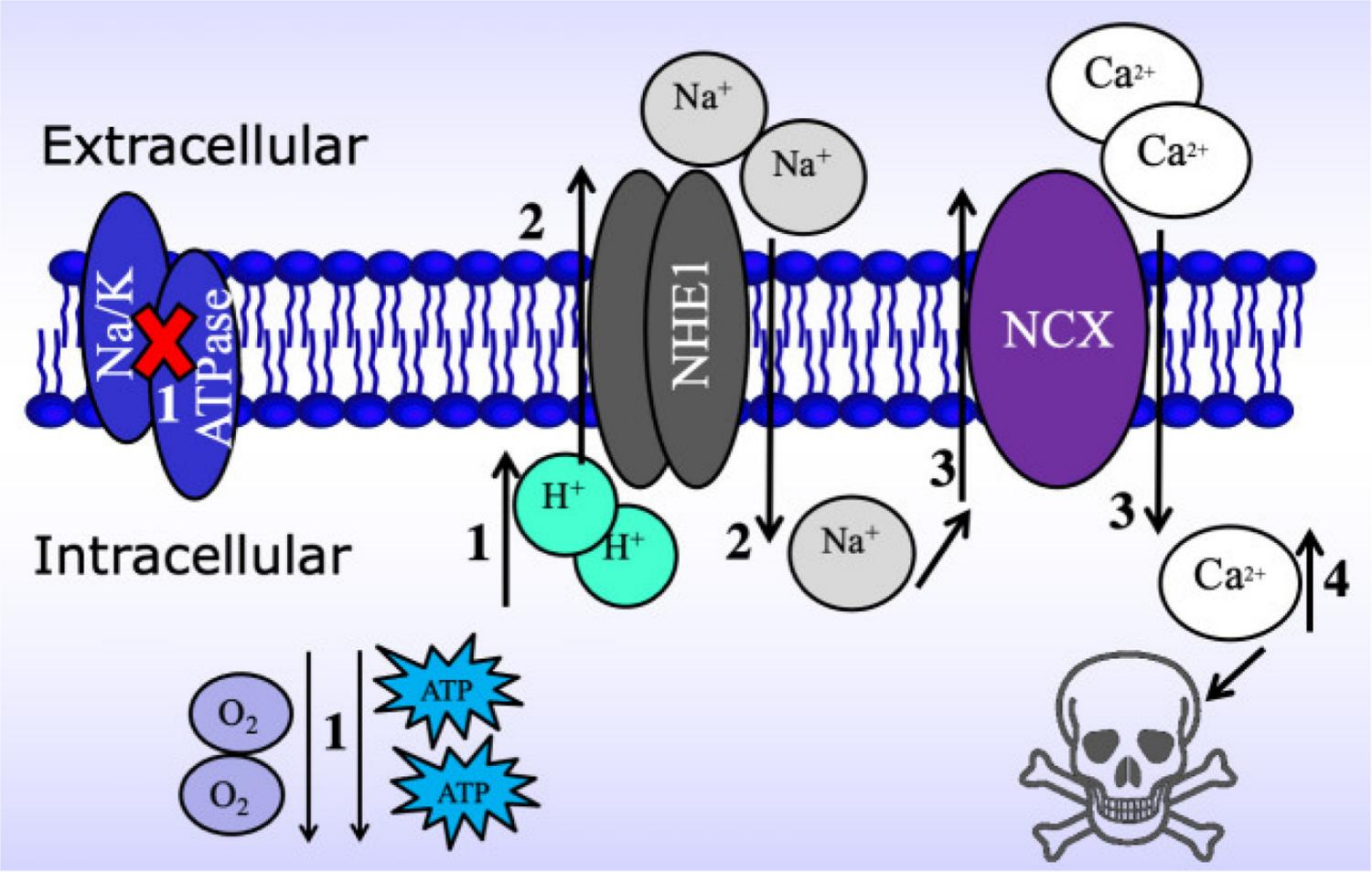
Schematic of the mechanism by which the Na + /H+ exchanger (NHE1) and the Na + /Ca2+ exchanger (NCX) interact to generate cardiac injury and dysfunction during ischemia/reperfusion challenge. (1). In the presence of reduced coronary blood flow and absence or reduced oxygen, ischemia causes a reduction in intracellular ATP levels and an accumulation of intracellular *H*+. The decreased levels of ATP stores impair Na + /K+ ATPase activity reducing Na+ export (2). The elevated intracellular *H*+ activates NHE1 to remove the *H*+ in exchange for extracellular Na+ (3). The increasing intracellular Na+ via NHE1, slows the removal of intracellular calcium by NCX or drives NCX in the reverse direction thereby increasing intracellular calcium concentrations in the cell (4). The excess entry of Ca2+ in the cell results in contractile dysfunction and structural damage to the myocardial cell. Republish from *Karmazyn M, Pierce GN, Fliegel L. The Remaining Conundrum of the Role of the Na* + */H+ Exchanger Isoform 1 (NHE1) in Cardiac Physiology and Pathology: Can It Be Rectified? Rev Cardiovasc Med. 2022 Aug 15;23*(8)*:284. doi: 10.31083/j. rcm2308284. PMID: 39076631; PMCID: PMC11266974*. This is an open access article under the CC BY 4.0 license.

Early‐generation NHE‐1 inhibitors (particularly the acylguanidine/guanidine class) were developed to limit I/R injury by preventing Na^+^ influx during acidosis. Clinical researches ve tested this hypothesis most prominently in high‐risk ischemic settings. Preclinical studies consistently demonstrate a recurring pattern: robust preclinical infarct‐sparing signals but mixed clinical efficacy, highly dependent on timing, dose, and patient subgroup [9] (Figure 3). In surgical ischemia, NHE‐1 inhibition was associated with reductions in peri‐procedural myocardial infarction in some analyses, whereas broader acute coronary syndrome or reperfusion settings did not show consistent benefit on clinical outcomes when pooled across heterogeneous populations. Evidence indicates that interpretation is complicated by endpoint selection, competing risks, and the narrow therapeutic window when NHE‐1 flux is maximally engaged by intracellular acidosis and Na^+^ loading [41, 42].

Eniporide inhibits NHE‐1 selectively, blocking Na^+^ uptake during ischemia and reperfusion. This prevents subsequent Ca^2+^ overload and cardiomyocyte death. Animal models showed substantial cardioprotection, prompting clinical trials in acute myocardial infarction [43, 44]. The phase II ESCAMI study tested four doses (50, 100, 150, and 200 mg) given intravenously over 10 min before reperfusion [44]. Stage 1 identified 100 and 150 mg as potentially beneficial based on enzymatic markers of infarct size. Stage 2 focused on these doses but found no significant reduction in infarct size or clinical outcomes [12]. Pharmacokinetics were linear with a half‐life of ~2 h and clearance of 30–35 L/h. Platelet assays confirmed NHE‐1 inhibition at 100–150 mg without excessive toxicity [45]. The negative results probably reflect poor timing rather than an invalid target. Eniporide must reach effective concentrations during early reperfusion when tissue acidosis peaks. Subgroup analysis hinted at benefit in late reperfusion patients (> 4 h from symptom onset) receiving 150 mg, suggesting that treatment windows and patient selection matter [12, 44].

Cariporide was evaluated in large clinical trials such as GUARDIAN and EXPEDITION. Although these did not meet primary endpoints across broad patient populations, subgroup analyses indicated reduced myocardial infarction rates in high‐risk surgical cohorts. However, increased cerebrovascular events with higher doses of cariporide dampened enthusiasm for widespread clinical use. The large GUARDIAN trial, which enrolled over 11,000 patients with acute coronary syndromes and high‐risk revascularization procedures, failed to show a significant reduction in the primary endpoint of death or myocardial infarction across treatment groups, although high‐dose cariporide was associated with reduced events in the subset undergoing coronary artery bypass grafting [46]. In the EXPEDITION study, cariporide reduced the early incidence of death or MI in CABG patients, but this cardioprotective signal was offset by an increased rate of cerebrovascular events and mortality, limiting clinical adoption [47].

### 5.2 Limitations and Safety Concerns in Clinical Translation

The major barrier to clinical adoption of NHE‐1 inhibitors is therapeutic index. Although NHE‐1 activates mainly during acidosis, systemic blockade affects tissues where the exchanger supports stress responses, ion homeostasis, and vascular regulation. High‐dose treatment produced cerebrovascular adverse events that offset cardioprotection in some trials, creating an unfavorable risk‐benefit profile [40, 41]. Two additional problems complicate interpretation. First, some NHE‐1 inhibitor scaffolds show growth‐inhibitory effects in tumor models independent of NHE‐1 blockade, indicating off‐target activity and poor chemical selectivity [48]. Second, NHE‐1 functions not only as a transporter but also as a signaling hub whose regulatory tail integrates kinase and phosphatase inputs. Blunt pore blockade disrupts both beneficial housekeeping functions and pathological signaling. This dual role has shifted attention toward strategies that modulate NHE‐1 regulation rather than eliminate transport entirely [21, 24].

### 5.3 Indirect Inhibition via SGLT2i: Mechanisms and Evidence

Beyond direct pharmacologic inhibition, SGLT2i produce large and reproducible reductions in heart‐failure events and kidney disease progression, stimulating intense interest in off‐target or downstream cellular mechanisms. One hypothesis held that SGLT2i directly inhibits NHE‐1, lowering intracellular Na^+^ and improving Ca^2+^ handling and mitochondrial function‐ a mechanism that could explain rapid cardiovascular effects disproportionate to glucose lowering [30, 49]. Direct NHE‐1 inhibition, however, remains controversial. Empagliflozin did not inhibit NHE‐1 across a wide concentration range [50]. Research data now distinguish direct pharmacologic inhibition from functional suppression through upstream pathways [49, 51]. SGLT2i benefits appear to arise from integrated effects on cellular stress rather than single‐target blockade. NHE‐1 modulation is a downstream consequence: SGLT2i attenuate the stimuli that upregulate NHE‐1 and thereby normalize exchanger activity indirectly [49].

Several mechanisms contribute to this indirect modulation. First, SGLT2i improves cellular energetics by inducing glucosuria and promoting a shift toward fatty acid oxidation and ketone utilization, which enhances ATP generation and reduces metabolic stress and intracellular acidosis [15]. Second, these drugs reduce oxidative stress and suppress inflammatory pathways such as the NLRP3 inflammasome, disrupting feed‐forward cycles of cellular injury and NHE‐1 upregulation. Third, systemic hemodynamic effects‐ including natriuresis, reduced preload and afterload, and lower myocardial wall stress‐ limit mechanical triggers of NHE‐1 activation while concurrent reductions in sympathetic tone and renin‐angiotensin‐aldosterone signaling further suppress stretch‐ and kinase‐mediated stimulation [52].

Several mechanisms contribute to this indirect modulation. SGLT2i improves cellular energetics through metabolic reprogramming. Glucosuria creates a mild energy deficit, shifting myocardial fuel preference toward fatty acid oxidation and ketone utilization. Ketones are more oxygen‐efficient than glucose, increasing ATP generation while reducing metabolic stress and intracellular acidosis [15]. SGLT2i also activate AMPK and sirtuins while suppressing mTOR signaling. This combination enhances autophagy and mitochondrial quality control, reducing mitochondrial dysfunction and ROS production. Less ROS means less activation of redox‐sensitive kinases that drive maladaptive NHE‐1 upregulation [15].

Systemic hemodynamic and neurohormonal effects provide an additional layer of indirect modulation of NHE‐1. The natriuretic and osmotic diuretic actions of SGLT2i reduce preload, afterload, and myocardial wall stress, which are key mechanical triggers of NHE‐1 activation. Concurrent reductions in sympathetic tone and renin‐angiotensin‐aldosterone system signaling further limit stretch‐ and kinase‐mediated stimulation of the exchanger. These integrated effects are highlighted as major contributors to the rapid and sustained benefits of SGLT2 inhibition in heart failure, independent of glycemic control [52].

To sum up, these data support a model in which SGLT2i restores a more favorable metabolic, redox, and hemodynamic environment, thereby reducing the pathological signals that necessitate NHE‐1 upregulation. In this context, NHE‐1 modulation reflects convergence of multiple protective pathways rather than direct pharmacologic targeting, explaining why it is increasingly positioned as one node within a broader ion‐homeostasis and stress‐response network.

## 6 Discussion

NHE‐1 represents a critical yet paradoxical cellular ion regulator that is simultaneously essential for physiological homeostasis and potentially destructive under pathological conditions. During ischemia, sustained NHE‐1 activity drives Na^+^ and Ca^2+^ overload, initiating a cycle where ionic dysfunction leads to further damage [8]. The exchanger's effects depend on context: modest activation protects cells during mild stress [53, 54], while excessive stimulation contributes to cell death. The core challenge remains developing interventions that selectively suppress pathological NHE‐1 activation without disrupting its essential homeostatic functions [9].

Clinical trials of NHE‐1 inhibitors have produced disappointing results. Cariporide and eniporide reduced infarct size in animal models but failed to show consistent benefit in patients [9, 10]. In some cohorts, cariporide increased stroke incidence, possibly from excessive dosing. SGLT2i appear to perform better, though whether they act through direct NHE‐1 inhibition remains disputed. Blaettner et al. found that both SGLT2i and NHE‐1 inhibitors produced similar cardioprotective effects in ischemia‐reperfusion models [55]. However, Chung et al. challenged direct NHE‐1 inhibition claims, suggesting the mechanisms are more nuanced [34, 50]. The key is that cardiac protection requires understanding NHE‐1 within a broader metabolic and hemodynamic context, rather than through simple, direct inhibition.

Next‐generation NHE‐1 inhibitors attempt to restrict drug action to diseased tissue through targeted delivery or conditional activation. Tissue‐selective strategies have shown promise in preclinical models: in pulmonary arterial hypertension, selective NHE‐1 blockade reduces right ventricular dysfunction without apparent systemic toxicity [56]. Similar approaches in cancer demonstrate that local NHE‐1 inhibition can restore immune function and reprogram tumor metabolism [1, 57]. Translating these findings to cardiovascular patients will require biomarkers that identify which patients have pathological NHE‐1 hyperactivity and which cardiac compartments are most affected. Future research must prioritize biomarker development and patient stratification to optimize these targeted interventions.

NHE‐1 inhibition remains difficult but not impossible. Early trials failed, probably from excessive doses, lack of tissue selectivity, and poor patient selection [9]. Three approaches attempt to solve these problems. New chemical scaffolds like pyrazinoyl guanidines may sidestep the off‐target effects that undermined cariporide and eniporide [58]. Advances in understanding NHE‐1 regulation now allow targeting of specific conformational states or regulatory domains rather than global pore blockade [21]. Context‐specific strategies exploit the spatial and temporal restriction of NHE‐1 hyperactivity to diseased tissue, enabling prodrug approaches or targeted delivery. The rationale for pursuing these strategies comes from consistent animal data showing that NHE‐1 blockade reduces pathological remodeling and slows heart failure progression [2]. Clinical success will require achieving the selectivity that first‐generation inhibitors lacked.

Structural and biophysical research has clarified that NHE‐1 activity depends not only on the transmembrane pore but also on a large cytosolic C‐terminal tail that integrates kinase and phosphatase inputs [21]. This tail converts stress signals into graded activation states rather than binary on‐off switching, allowing proportional responses to acidosis, mechanical stretch, and oxidative stress. ERK1/2‐mediated phosphorylation at specific serine and threonine residues relieves autoinhibitory constraints and stabilizes active conformations [59], producing a continuum of activity states. Hendus‐Altenburger et al. demonstrated that calcineurin selectively dephosphorylates Thr779, inducing distinct functional states without suppressing basal activity [58]. This suggests that pathological hyperactivation reflects sustained occupancy of specific phosphorylation states. Therapeutic strategies could disrupt kinase‐tail interactions [60], stabilize autoinhibited conformations, or bias phosphorylation equilibrium toward less active states. Beyond phosphorylation, Ca^2+^‐sensitive allosteric control through calmodulin and calcineurin homologous proteins provides additional regulatory complexity. Köster et al. showed that Ca^2+^‐loaded calmodulin engages NHE‐1 through multiple binding modes, functioning as a flexible allosteric regulator rather than a simple activator [24]. Targeting regulatory sites rather than the transport pore may achieve the specificity and safety that earlier inhibitors lacked.

Tissue‐targeted delivery represents another approach to restrict NHE‐1 suppression to cardiovascular compartments where pathological hyperactivation is most prominent while minimizing systemic effects [2, 41]. NHE‐1 hyperactivity couples tightly to localized biochemical conditions, including acidosis, oxidative stress, and sustained kinase signaling, features spatially restricted to pathological niches such as ischemic border zones, hypertrophied myocardium, and pulmonary vascular lesions. These endogenous activation signals could be leveraged by conditionally active compounds or targeted delivery systems. Rimeporide ongoing development in Duchenne muscular dystrophy and pulmonary arterial hypertension illustrates how restricting NHE‐1 inhibition to disease contexts with chronic ionic stress may offer favorable risk‐benefit ratios compared to acute coronary syndromes [56], [64].

Effective NHE‐1 modulation will likely require combining regulatory‐site targeting, tissue‐specific delivery, and disease‐state gating rather than uniform systemic exposure. Achieving selectivity through mechanism rather than potency represents the critical distinction between first‐generation failures and emerging therapeutic strategies. Whether these approaches translate to clinical benefit will depend on rigorous patient stratification, biomarker‐guided dosing, and careful attention to the lessons from earlier trials.

## 7 Conclusions

NHE‐1 inhibition still has therapeutic potential, but the path forward is not straightforward. Cariporide and eniporide failed due to systemic blockade caused unacceptable toxicity at clinically relevant doses. Current research focuses on restricting inhibition to diseased tissue through regulatory‐site targeting, selective delivery, or conditional activation. Clinical translation will require biomarkers to identify which patients have NHE‐1‐driven disease and pharmacological tools more selective than earlier compounds.

## Data Availability

Journal of Cellular Physiology (JCP).
